# Ring artifact reduction via multiscale nonlocal collaborative filtering of spatially correlated noise

**DOI:** 10.1107/S1600577521001910

**Published:** 2021-04-16

**Authors:** Ymir Mäkinen, Stefano Marchesini, Alessandro Foi

**Affiliations:** a Tampere University, Finland; b SLAC National Accelerator Laboratory, 2575 Sand Hill Road, Menlo Park, CA 94025, USA

**Keywords:** BM3D, noise, image processing, tomography, artifact

## Abstract

X-ray micro-tomography systems often suffer severe ring artifacts in reconstructed images. These artifacts are caused by defects in the detector, calibration errors, and fluctuations producing streak noise in the raw sinogram data. In this work, these streaks are modeled in the sinogram domain as additive stationary correlated noise upon logarithmic transformation. Based on this model, a streak removal procedure is proposed where the Block-Matching and 3-D (BM3D) filtering algorithm is applied across multiple scales, achieving state-of-the-art performance in both real and simulated data. Specifically, the proposed fully automatic procedure allows for attenuation of streak noise and the corresponding ring artifacts without creating major distortions common to other streak removal algorithms.

## Abstract 2

Here, streak noise is modeled in the sinogram domain as correlated noise, which is attenuated by the Block-Matching and 3-D (BM3D) filtering algorithm applied across multiple scales. The proposed procedure is fully automatic and attenuates streak noise and the corresponding ring artifacts without creating major distortions common to other streak removal algorithms.

## 1. Introduction   

Ring artifacts are ubiquitous in computed tomography (Jha *et al.*, 2013 ▸; Artul, 2013 ▸; Boas & Fleischmann, 2012 ▸); they originate from angular streak noise in measured raw sinogram data used to reconstruct a tomographic volume (Croton *et al.*, 2019 ▸) and appear as darker or lighter circles or arcs centered on the axis of rotation for data acquisition. Streak noise can be caused by mis-calibration of detector linear response, beam fluctuations, beam hardening, or dusty or damaged scintillator screens (Haibel, 2008 ▸; Vidal *et al.*, 2005 ▸; Anas *et al.*, 2010 ▸).

Minimization of ring artifacts by using adequate scanning protocols (Pelt & Parkinson, 2018 ▸), high quality scintillator screens and detectors is possible. It is, however, difficult to completely avoid such artifacts and therefore achieve highest quality reconstruction solely by experimental measures. Several algorithms have been proposed to reduce ring artifacts in tomographic imaging, including wavelet-FFT filters (Münch *et al.*, 2009 ▸), combinations of polynomial smoothing filters and careful calibration of the detector response function (Vo *et al.*, 2018 ▸; Croton *et al.*, 2019 ▸), or iterative algorithms (Paleo & Mirone, 2015 ▸) that combine regularized reconstruction with denoising.

In this work, we model the streak noise as a spatially correlated noise in the sinogram domain, and propose a denoising procedure aiming to remove the streak noise before reconstruction. The denoising procedure is based on collaborative filtering, which employs both non-local self-similarity and transform-domain shrinkage to denoise a noisy signal through jointly transformed grouped blocks. In particular, we use the image denoising algorithm BM3D (Dabov *et al.*, 2007 ▸, 2008 ▸), leveraging the recent inclusion of exact transform-domain noise variances (Mäkinen *et al.*, 2020 ▸), which allow for accurate modeling of long noise correlation within the jointly transformed blocks.

Noting that some streaks may be too wide to be adequately captured by a group of standard-sized BM3D blocks, we further propose multiscale streak removal with BM3D. The proposed procedure is fully automatic and includes self-calibration of the filtering strength. We demonstrate the superior performance of the proposed approach on real data from the table-top Prisma XRM microCT at Sigray, and from the synchrotron-based microCT at the Advanced Photon Source (APS) in Argonne, available through Tomobank (De Carlo *et al.*, 2018 ▸).

## 2. Transform-domain collaborative filtering of correlated noise   

In this section, we interpret sinogram streaks as spatially correlated noise, formalizing streak removal as filtering of correlated noise where the streaks follow a basic stationary model. As a powerful tool to deal with this model, we adopt a recent BM3D designed for dealing with long-range correlation such as that which characterizes the streaks. This constitutes the denoising module at the core of a multiscale and nonstationary filtering architecture that will be presented in Section 3 for the more general case of real-world streak noise.

### 2.1. Correlated noise model   

We consider a noisy input 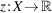 to be a combination of underlying data *y* and additive stationary spatially correlated noise η to be filtered, 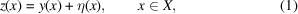
where 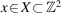 is the coordinate in the finite two-dimensional image domain *X* (representing angles and displacements when *z* is a sinogram) and 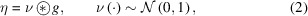
with ν being the zero-mean independent and identically distributed (i.i.d.) Gaussian noise with unit variance, and ‘
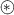
’ denoting 2-D convolution with the kernel *g*. The kernel *g* defines the spatial correlation of the noise as well as the noise strength, with 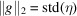. An equivalent way of representing correlated noise is by its power spectral density (PSD) Ψ, 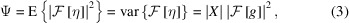
with 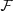 being the 2-D Fourier transform and |*X*| denoting the cardinality (*i.e.* number of elements) of *X*. Equivalently, a kernel *g* satisfying (2)–(3) can be defined from Ψ as 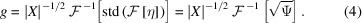



#### 2.1.1. Basic model for constant stationary streaks   

**Figure 1:**
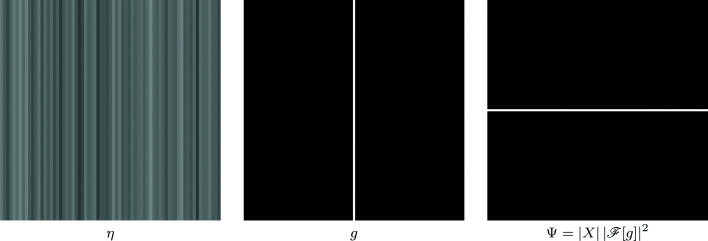
Example noise 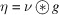, 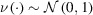, the corresponding correlation kernel *g*, and PSD Ψ. For the kernel and the PSD, black pixels of the image correspond to value 0 in the data.

**Figure 2:**
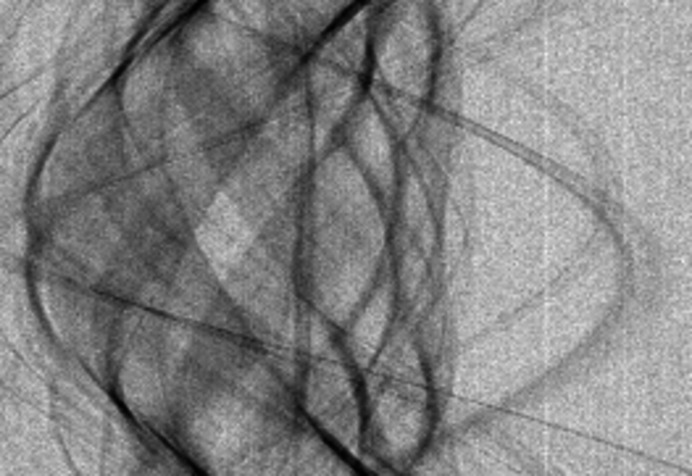
Example of streak noise in a sinogram (a fragment of *Fly*) that can be seen as well approximated by the model in Figure 1 ▸.

Although the streaks are originally multiplicative in nature, sinogram data are considered upon a logarithmic transformation and therefore the streaks can be modeled by the additive noise η in (1). The sinogram streak noise is fairly constant in the angular dimension, presenting very long-range correlation in the noise along this dimension. Treating angle as the vertical dimension and displacement as horizontal, we consider the basic case of horizontally white and vertically constant streak noise. Such noise can be modeled through (2), when setting *g* as a vertically constant line. This simple kernel as well as the corresponding PSD Ψ are demonstrated in Figure 1 ▸; an example of real streak noise viably approximated through this model is shown in Figure 2 ▸.

In practice, the above simple model cannot be used but for small segments of the sinograms, as streak noise can often feature horizontal correlation, vertical variations, or nonstationarities that are not described by the model. In Section 3, a complete processing pipeline is further proposed to allow modeling more complex cases of streak noise through (1)–(4), enabling their attenuation through the collaborative filter.

### 2.2. Transform-domain collaborative filtering and BM3D   

The rationale of transform-domain filtering is to work with a representation of the signal where most of the signal is compacted to only a few coefficients, whereas the remaining coefficients mostly comprise noise. Hence, by attenuating the coefficients with a non-linear shrinkage operator, it is possible to attenuate noise while keeping most of the signal intact. Nonlocal collaborative filters utilize this property in the context of collective transform coefficients of groups of similar blocks extracted from the image. In all of the following sections, we consider the recently proposed variant (Mäkinen *et al.*, 2020 ▸) of BM3D for correlated noise denoising where the input is *z* and the goal of denoising is to estimate *y* based on the statistics of η or equivalently knowledge of Ψ or *g*.

In BM3D, all operations are made with regard to a reference block moving through the image. For each position of the reference block, the following steps are executed:

(i) Collect similar blocks into a group through **block-matching**.

(ii) Obtain the 3-D transform spectrum by collectively transforming the obtained blocks.

(iii) Perform **shrinkage**.

(iv) Transform the shrunk spectra back to block estimates and **aggregate** them to the original locations from which they were collected.

**Figure 3:**
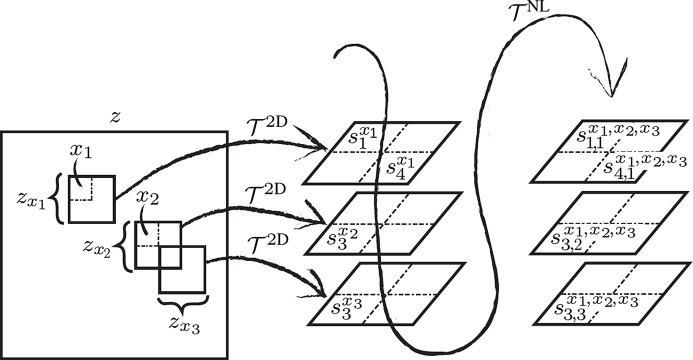
Notation and indexing of patch coordinates *x*_*l*_, patches 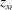, and coefficients 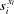 and 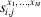 in the the corresponding 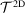 spectra and 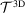 spectrum, reproduced with permission from Mäkinen *et al.* (2020 ▸). The illustration is for a group of three blocks of size 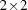 at coordinates 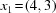, 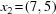, 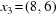 within a 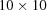-pixel image.

The 3-D transform spectrum of the grouped noisy blocks is obtained through first applying a 2-D transform 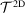 locally to each block, then a 1-D transform 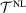 through the ‘stack’ of grouped blocks. Denoting by 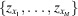 a group of *M* blocks of *N* pixels extracted from *z* at coordinates *x*_1_,…, *x*_*M*_, we obtain the 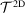 spectrum coefficients as 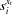 = 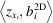, for *i* = 1,…, *N, t* = 1,…, *M*, where 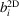 is the *i*th basis function of 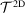. The 3-D spectrum coefficients are calculated through the direct tensor product of the 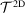 and 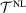 transforms, as 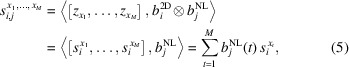
where 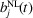 is the *t*th element of the *j*th basis function 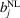 of 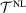, ⊗ denotes the tensor product, and 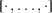 denotes the stacking of the blocks along the third dimension. The indexing and notation of the transform spectrum coefficients are illustrated in Figure 3 ▸.

In each step of the algorithm, the variances of 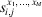 play a key role; we denote them by 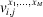. For their calculation, we refer the reader to Mäkinen *et al.* (2020 ▸) ▸. Here, we provide a summary of the macroscopic operations of the algorithm.

#### 2.2.1. Block-matching   

For each reference block, BM3D defines a local neighborhood from which similar blocks are collected. Each block in the neighborhood is ranked by 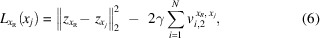
where 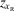 is the reference block, 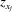 is a potential match, 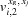 is the *i*th coefficient of the block-pair transform-domain variance corresponding to block difference, and 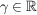. The common aim of block-matching is to find blocks which are the most similar to the reference block in terms of the underlying noise-free content. When only a noisy image is available, the similarity is evaluated between noisy blocks and the term scaled by γ in (6) compensates for bias in the ranking caused by noise correlation. Specifically, with 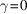 the matches would be mainly guided by the strong vertical correlation of the streak noise and thus be located along the streaks, largely ignoring any similarity of the underlying signal; setting 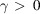 mitigates this bias by promoting matching of blocks in which the noise is not correlated with that of the reference block. In particular, we employ 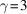 as proposed by Mäkinen *et al.* (2020 ▸) ▸ for the general case of correlated noise, facilitating further the matching of blocks which differ from the reference block mainly due to the variance of the block difference.

The common design of BM3D includes two distinct stages of denoising with different shrinkage operators, meaning that the full image is processed twice. In the second stage, the block-matching is commonly executed on the image estimate produced by the first denoising stage. As this image can be presumed noise-free, the second block-matching is executed without any compensation for noise correlation.

#### 2.2.2. Shrinkage of the 3-D spectra   

The core of BM3D is shrinkage performed on the 3-D transform spectrum of the grouped noisy blocks. For a given transform-domain coefficient of the group, a generic shrinkage can be expressed as 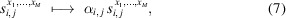
where α_*i*, *j*_ is a shrinkage attenuation factor which depends on 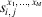, the noise statistics, and possible other priors.

BM3D utilizes two shrinkage operations: in the first denoising stage, the denoising process performs shrinkage by hard-thresholding; the second stage employs a Wiener filter, utilizing the hard-thresholding image estimate as a pilot signal.

In hard-thresholding, the shrinkage is performed by setting spectrum coefficients smaller than a threshold to zero, as they are mostly composed of noise,
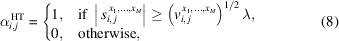
where 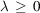 is a fixed constant.

In Wiener filtering, the attenuation coefficients of the transfer function are computed from the previous estimate, used as pilot signal, and from the variance of the noise spectrum coefficients as 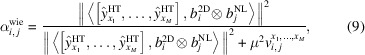
where 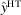 is the estimate of *y* obtained from the hard-thresholding stage, and μ^2^ is a scaling factor included due to aggregation to influence the bias-variance ratio we wish to minimize through the Wiener filter.

#### 2.2.3. Aggregation   

After calculating the attenuation factors of the group, they can be applied to the 3-D transform spectra to obtain estimates for the grouped blocks,
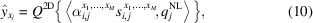
where *Q* ^2D^ is the inverse transform of 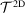, and 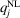 is the *j*th transform basis function of the inverse of 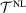.

Hence, an estimate is produced for all blocks included in the group. As a new group is built for every position *x*_R_ of the reference block, there is a large amount of block estimates providing a highly redundant covering of the image. Let *X*_R_ be the set of coordinates of all reference blocks and denote by 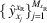 the set of estimates (10) for the group of blocks matched to the reference block at position *x*_R_. Then, the block estimates of an image are 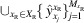 and they can all be distinct.

We aggregate all block estimates at their respective positions into the image through an adaptive weighted average,
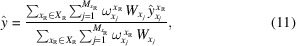
where 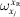 is a block-specific weight and 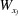 is a windowing function over blocks at position *x*_*j*_. The weights 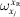, inversely proportional to the residuals of transform-domain noise variances, promote estimates with less residual noise to improve the quality of the final estimate.

**Figure 4:**
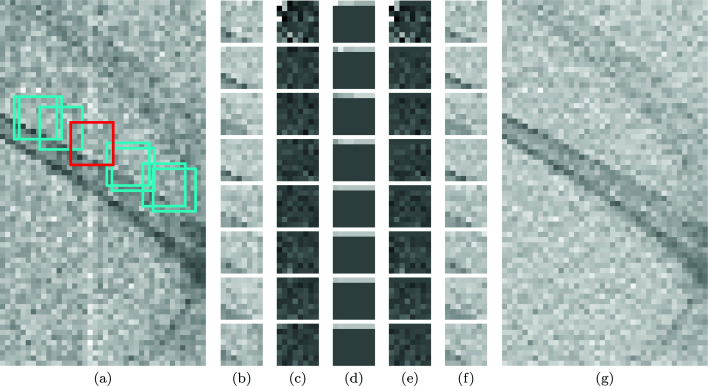
Denoising a part of *Fly* (a portion of Figure 2 ▸) with vertical streak noise with Ψ as in Figure 1 ▸. Left to right: (*a*) positions *x*_1_,…, *x*_8_ of one group of blocks with reference block in red; (*b*) contents of the eight matched blocks 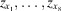; (*c*) the resulting 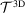 spectrum coefficients 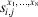; (*d*) corresponding 3-D noise standard deviations 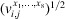; (*e*) hard-thresholded coefficients 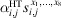; (*f*) the denoised group of blocks 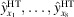, and (*g*) the denoising result of hard-thresholding 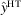. For the spectrum coefficients and the standard deviations (*c*, *d*, *e*), 50%-gray pixel color in the figure corresponds to value 0 in the data.

The steps for denoising a group of blocks are demonstrated in Figure 4 ▸.

## 3. Processing pipeline   

In this section, we consider the necessary steps for modeling the streak noise through (1) for real sinogram data, hence allowing the effective application of BM3D for streak removal.

### 3.1. Bright-fielding and log-transformation   

The optical attenuation through the sample is determined experimentally via bright-field corrections requiring two additional inputs: the bright-field and the dark-field (Seibert *et al.*, 1998 ▸). The bright-field is an acquisition obtained by the imaging procedure with no sample, and the dark-field is obtained with no beam; both are 2-D arrays the size of effective pixels of the detector. Furthermore, the Beer–Lambert law relates the X-ray transform through the sample to the optical attenuation by a logarithmic transformation (Swinehart, 1962 ▸).

Hence, the raw projections *P*_raw_ are first normalized as 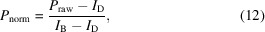
where *I*_D_ is the dark-field and *I*_B_ is the bright-field,^1^ and then log-transformed as 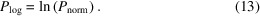
Bright-fielding (12) provides a partial, but not thorough, correction of the streak noise (Davidson *et al.*, 2003 ▸); the denoising pipeline of the following sections is designed to attenuate the remaining streak noise.

#### 3.1.1. Noise in the projections   

Apart from possible completely defective detectors^2^ we treat the variation in detector response as normally distributed. We further model the streak noise as locally stationary, meaning that the streak variance is presumed constant within sufficient area (*i.e.* the block-matching search neighborhood) for the application of BM3D. As the data are obtained through a photon-counting detector, the statistics of the measured raw data can be further modeled through a Poisson distribution. Considering both the approximately normally distributed streak noise and the Poissonian component, noise in projections normalized by (12) can be modeled as 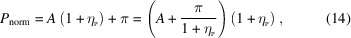
where *A* are the noise-free projections, 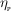 is the normally distributed streak noise component, and π is (approximately) white Poissonian noise with zero mean; all components of (14) are considered as 3-D arrays and multiplications are elementwise.

We note that the natural logarithm of (13) acts as a variance-stabilizing transformation for the multiplicative noise component 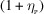. Hence, we have 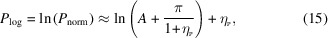
where the approximation comes from 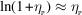. The additive noise component 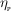 in (15) corresponds to the streak noise to be denoised. As here we only aim to attenuate the streak noise, through denoising we estimate 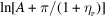; the embedded noise term 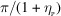 although not i.i.d. is nevertheless (approximately) white and does not present streaks.

Individual sinograms, each of which is defined as a cross section of the stack of projections *P*_log_, are denoted as 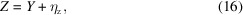
where *Y* denotes the underlying streak-free sinogram, and 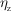 is the corresponding cross section of 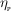. The sinograms *Z* are used as the input for the processes in the following Section 3.2.

### 3.2. Multiscale filtering architecture   

In the following, we assume that sinograms *Z* are oriented such that streaks are oriented vertically, *i.e.* the angular component is vertical and the displacement is horizontal.

The streak noise 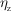 is characterized by very long-range correlation. In particular, because vertically there are no high-frequency streak noise components, the streaks can be filtered entirely at a *coarse vertical scale*, with consequent benefits in terms of efficacy and computational efficiency. Furthermore, BM3D operates using blocks of fixed size within a limited neighborhood which may be too small to fully denoise the wider streaks. Thus, we also want to denoise across *multiple horizontal scales* to effectively attenuate streaks of varying sizes.

**Figure 5:**
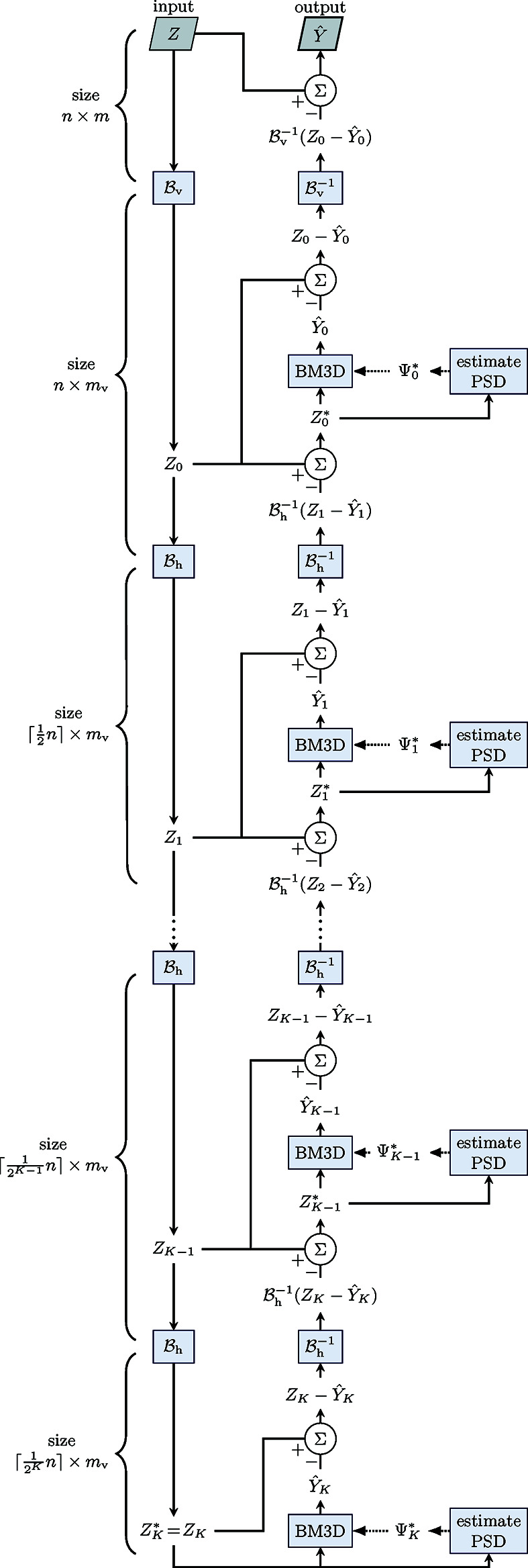
Flowchart of the multiscale denoising process, starting from the noisy sinogram *Z* and resulting in the estimate 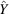 of the streak-free sinogram, both of size 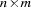. First, *Z* is vertically rescaled into a sinogram *Z*_0_ of size 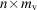, 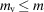, through the binning operator 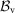. Then, by repeated horizontal binning 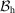, *Z*_0_ is progressively downscaled into a series of sinograms 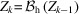, 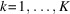, each of size 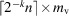. The coarsest scale noisy input 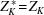 is denoised with BM3D to produce 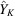. Then, recursively for 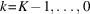, the noisy input 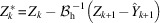 is denoised by BM3D to produce 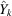; this definition of 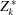 means that the coarse-scale horizontal components of *Z*_*k*_ are replaced by 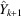. The PSD for each scale is estimated as described in Section 3.3.2. The resulting estimate 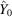 of the horizontal debinning (size 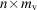) similarly replaces the coarse-scale vertical components of *Z* to obtain the full-size estimate 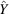.

Our multiscale implementation is based on a simple and efficient pixel binning to go towards coarser scales by replacing adjacent pixels by their sum. To go back towards finer scales we leverage the iterative debinning approach from Azzari & Foi (2016 ▸) ▸, which is based on spline upsampling. The multiscale denoising process is illustrated in Figure 5 ▸ and proceeds as follows.

We begin with a single *vertical* binning of the full noisy sinogram *Z* of height *m* to a sinogram *Z*_0_ of height 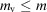 through a binning operator 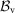. On *Z*_0_, we perform all consequent horizontal operations and denoising.

After vertical binning, the sinogram *Z*_0_ is progressively halved in size *K* times through a *horizontal* binning operator 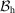: 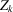 = 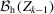 = 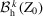 = 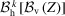, 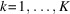. Denoting by *n* the width of *Z* and *Z*_0_, *Z*_*k*_ has width ⌈2^−*k*^ *n*⌉; with every binning, the streak width also gets halved. The multiscale denoising is operated in a coarse-to-fine fashion, where progressively for each 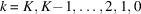 we obtain an estimate 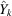 of 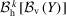. We start by taking as noisy input 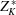 of BM3D the smallest binned sinogram *Z*_*K*_; in this way, we obtain from 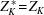 the coarsest estimate 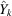, which is taken as initialization for the following recursive steps executed for each scale 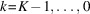:

(1) Replace the horizontal coarser-scale components of *Z*_*k*_ by those of the estimate 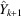, 
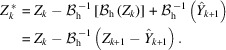
(2) Denoise 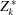 with BM3D to produce the estimate 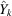.

**Figure 6:**
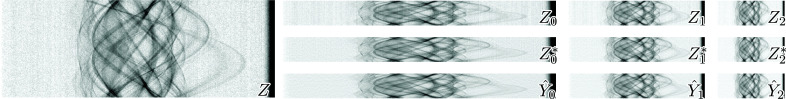
Multiscale denoising of *Fly*. Left: the noisy sinogram *Z*. Center and right: three scales of the multiscale denoising process, each showing *Z*_*k*_, 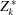, and 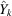. The full-size estimate 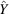 is displayed in Fig. 12 ▸.

The result 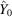 of the last denoising step is the fully denoised estimate the size of *Z*_0_. To produce the full-size estimate 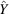 of *Y*, we replace the vertical coarse-scale components of *Z* with those of 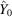, similar to the Step (1) above, 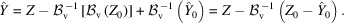
Figure 6 ▸ illustrates the sinograms over the various stages of the multiscale denoising process.

### 3.3. Multiscale noise model   

For BM3D denoising, we regard 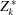 of each scale *k* as *z* of the model (1), as 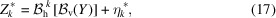
where 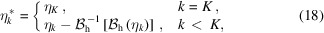
and 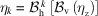.

This definition for 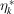, 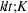, follows from considering the coarser-scale estimate 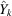 as perfectly denoised. Similar to (3), 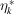 is treated as correlated noise with PSD
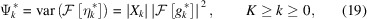
where 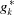 is a correlation kernel and 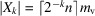. As per (4), the kernel 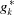 can be defined as 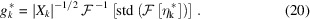



#### 3.3.1. Multiscale PSD of white streak noise   

Let 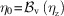 be horizontally white and vertically constant streak noise like in Figure 1 ▸. Under this assumption for η_0_, we have that also all 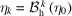 for 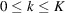 are horizontally white and vertically constant, with variance 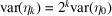. The doubling of the variance with every horizontal binning follows from the noise whiteness, which means that each pixel of the coarser scale sinogram is a sum of two pixels with independent noise of equal variance. Therefore, disregarding the specific support size of their actual finite realizations, we can identify these stationary random fields as 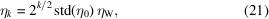
where η_W_ is a white streak noise like in Figure 1 ▸ with 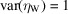. We can hence rewrite (18) as 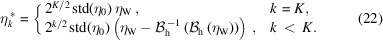
This together with (20) means that we can characterize 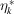 through a kernel 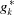 obtained by scaling either of two basic two kernels *g*_c_ and 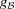, 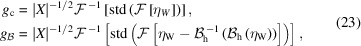
by a factor 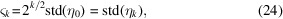
as 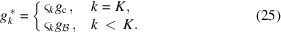
We note that although the equalities (23) formally depend on the realization size |*X*|, in practice this term only renormalizes the kernel with regard to the Fourier transforms; hence (23) can be computed for an arbitrary support.

**Figure 7:**
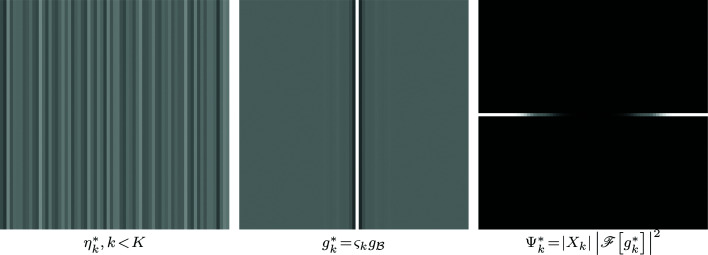
Example of noise 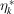 of 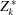, 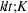, the correlation kernel 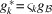 where 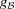 is produced by 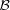 and 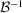, and the corresponding PSD 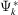. For the kernel, 50%-gray pixel color in the figure corresponds to value 0 in the data; for the PSD, black is 0. Note the missing low frequencies at the center of the PSD, and the higher-frequency nature of 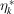 compared with the white streak noise η in Figure 1 ▸.

The kernel *g*_c_ is single-pixel wide and vertically constant like in Figure 1 ▸, with 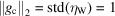. Example noise 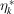, 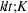, and the corresponding kernel 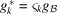 and PSD 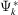 (19) are shown in Figure 7 ▸.

**Figure 8:**
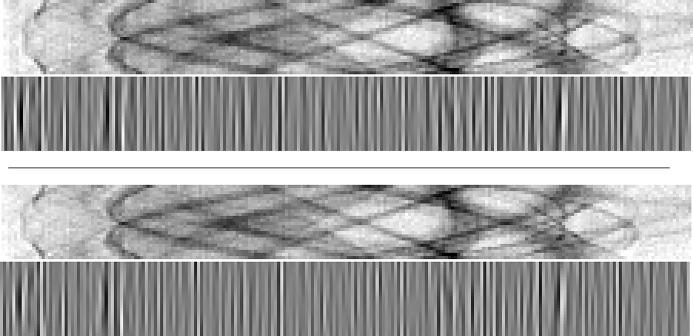
A fragment of *Z*_0_ of *Fly* and the corresponding 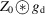 (top), and a fragment of 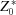 with the corresponding 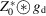 (bottom). For 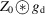 and 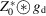, 50%-gray pixel color in the figure corresponds to value 0 in the data. Note how most of the signal of the sinogram fragments is not present in the convolved arrays, facilitating the estimation of the streak noise statistics. Although 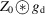 and 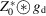 look very similar, careful visual inspection reveals slight differences similar to those between 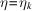 in Figure 1 ▸ and 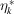 in Figure 7 ▸.

*Estimation of ς_0_.* To estimate 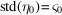, we first convolve *Z*_0_ with a 2-D kernel 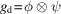 where ϕ is a 1-D column Gaussian function of length *m*_v_/2 and standard deviation *m*_v_/12 and ψ is a horizontal high-pass Daubechies wavelet ‘db3’ of length 6, hence convolution with *g*_d_ realizes low-pass filtering in the vertical and high-pass filtering in the horizontal. Thus, compared with *Z*_0_, 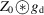 offers a lower signal-to-noise ratio (SNR), which facilitates the estimation of noise statistics; an example of *Z*_0_ and the corresponding 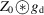 are shown in Figure 8 ▸ (top). One can compute an estimate of the standard deviation of 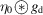 via its median absolute deviation (Hampel, 1974 ▸), 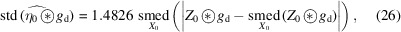
where smed denotes the sample median and the factor 1.4826 calibrates the estimate with respect to a normal distribution of the noise. As 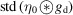 = 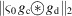, an estimate 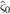 of ς_0_ can be obtained through 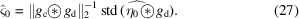



#### 3.3.2. Adapting the model to non-white streak noise   

The above model (21)–(25) assumes that the streak noise 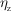 is horizontally white and stationary; however, real streak noise is never exactly white across the displacement, and may thus have significant differences in noise power between scales.

To adapt to these deviations from the model, we relax the definition (24) of ς_*k*_ and allow the scaling parameter 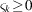 to vary with each scale *k*, while assuming the kernels as in (25) for simplicity.^3^ In this way, to adaptively model the PSD (19), we require only the estimation of ς_*k*_ on each 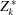.

*Estimation of ς_*k*_, 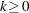.* For 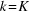, ς_*K*_ can be estimated from 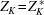 by trivial substitutions of 0 with *K* in (26) and (27). Although also for 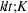 one could estimate ς_*k*_ similarly from *Z*_*k*_, a more accurate estimate can be obtained using 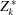 as this leverages the denoising of the coarser scales and thus 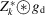 offers an even lower SNR than 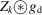. An example of 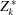 and the corresponding 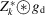 are shown in Figure 8 ▸ (bottom). Similar to (26) and for any 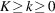, the standard deviation of the noise in 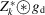 can be estimated as 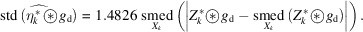
Noting that 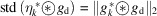, we then estimate ς_*k*_ as 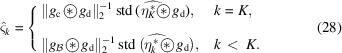



#### 3.3.3. Horizontal nonstationarity of η__Z__   

Variance of the streak noise may differ across the sinogram due to changes in photon flux or noise in the bright-field. Thus, it may not be possible to denoise 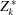 assuming a constant ς_*k*_ for all spatial positions without either oversmoothing or leaving noise artifacts in some areas. To adapt to horizontal nonstationarity, we further relax the model allowing ς_*k*_ to vary within each scale *k*. In particular, before noise estimation and denoising, we split 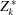 and 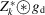 into overlapping, full-height segments. We apply BM3D separately on each segment of 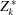, using a PSD scaled by 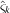 estimated on the corresponding segment of 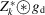, *i.e.* we consider each segment as a separate noisy image *z* with a corresponding Ψ. After denoising, the segment estimates produced by BM3D are recombined with a windowing function to form the full estimate 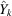.

### 3.4. Attenuation of extreme streaks   

We note that the projections often include several streaks caused by defects in the scintillator. These streaks can be far stronger than what are reasonably produced by the distribution of 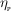 and therefore require a specific pre-processing. To this end, after applying the bright-field and before the multiscale denoising process, we run a simple procedure on *P*_log_ which aims to detect and attenuate only the most extreme streaks. First, we calculate the median across the angular dimension of the 3-D stack of projections as
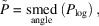
resulting in a 2-D map in which the streaks present as pixels extremely brighter or darker than their surroundings. To detect extreme outliers, for each coordinate *x* representing a single pixel of the detector and hence of 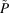, we fit a bivariate cubic polynomial ℘_*x*_ to a window 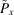 of 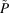 centered at *x*. Then, consistent with Gaussian modeling of 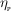, we mark the center pixel 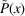 defective if 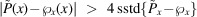, where sstd denotes the sample standard deviation; each marked pixel in 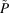 corresponds to a full column of the sinograms.

Each pixel of a defective column is replaced with the median of non-defective pixels within a 2-D window considering the displacement dimensions around it. We note that columns corrected in this way are unlikely to be completely free of streak noise; instead, the aim is to introduce less extreme pixel values that can be further denoised by the following applications of BM3D. In order not to overload the notation, we denote the output of this step as *P*_log_ identical to its input.

**Figure 9:**
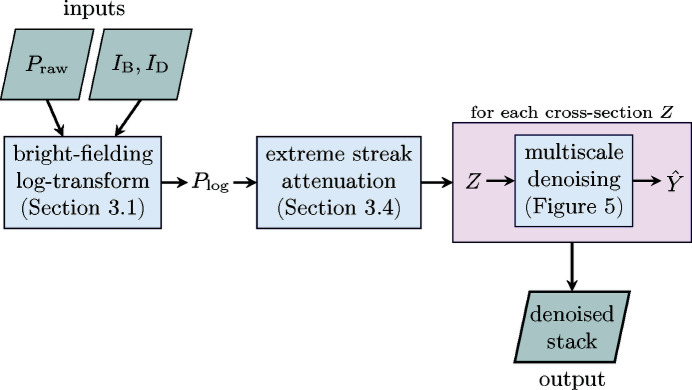
The complete denoising process, requiring as inputs the noisy projections *P*_raw_ and the bright- and dark-fields *I*_B_, *I*_D_, and producing as the output an estimate of the streak-free stack of projections composed of sinogram estimates 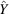.

The complete streak noise attenuation procedure is illustrated in Figure 9 ▸. The procedure is fully automatic, requiring as an input only the raw projections and the bright- and dark-fields.

## 4. Experiments   

We test our pipeline on synthetic data as well as two real acquisitions displaying ring artifacts. As a comparison, we show results for two leading streak-removal procedures from the Tomopy library (Gürsoy *et al.*, 2014 ▸): Münch *et al.* (2009 ▸) and Vo *et al.* (2018 ▸) ▸. In particular, for the latter we combine ‘Algorithm 3’, ‘Algorithm 5’, and ‘Algorithm 6’, which is demonstrated by Vo *et al.* (2018 ▸) ▸ to attenuate a variety of different streaks.

**Figure 10:**
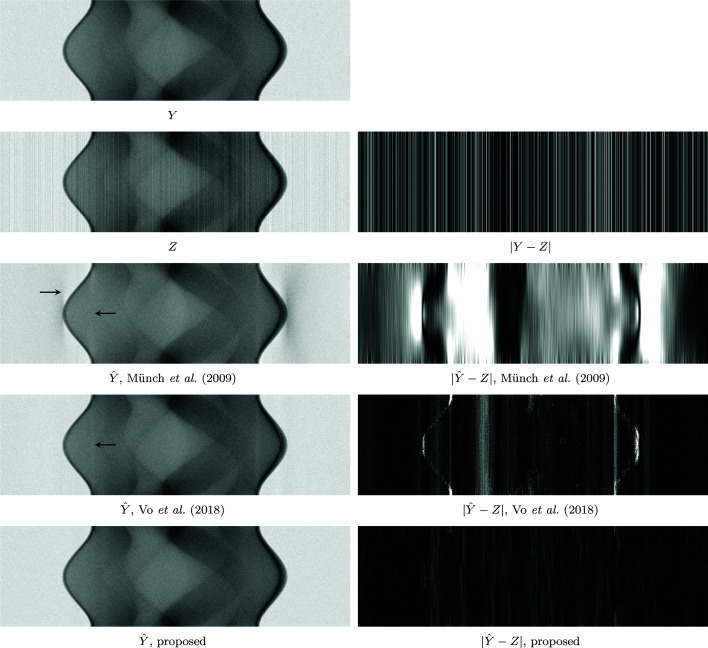
Left: comparison of sinograms after different denoising procedures on the Shepp–Logan phantom with noise as in (14) with 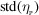 = 0.02 and signal peak 2560. Top to bottom: 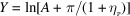; *Z*; Münch *et al.* (2009 ▸) ▸; Vo *et al.* (2018 ▸) ▸; proposed procedure based on BM3D denoising. Right: corresponding estimation errors. Note how both of the comparison methods create strong artifacts around the areas with the highest contrast, as pointed by the arrows. These artifacts are not present in either the noisy image *Z* or the BM3D-based estimate.

For the synthetic experiments, we use a sinogram (627 × 180 pixels) of the Shepp–Logan phantom obtained through MATLAB Radon transform upon a sign change and an exponential transformation. We regard this sinogram as the noise-free projections *A* and generate noise according to (14) with *g* as a one-pixel wide image-height vertical kernel like the one in Figure 1 ▸. To obtain streak noise of different strengths, the streak noise component 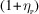 is generated with 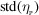 = 0.005, 0.01, 0.02, 0.05. Next, to generate noisy measurements with different SNR levels for the Poisson component, we separately scale *A* to the ranges [1280, 2560] (higher SNR) and [640, 1280] (lower SNR) and generate a Poisson variate with mean and variance 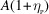, thus defining the Poissonian noise π as the difference between this Poisson variate and 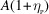. Furthermore, we include experiments with 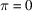 (infinite SNR), thus resulting in a total of 12 combinations of Poisson and streak noise strengths. We do not simulate extreme streaks. As the underlying data consist of only a single sinogram, we have 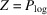 and we consider 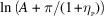 as the streak-free sinogram *Y*. The results of the phantom experiments are collected in Table 1 ▸, and illustrated in Figures 10 ▸ and 11 ▸.

**Figure 12:**
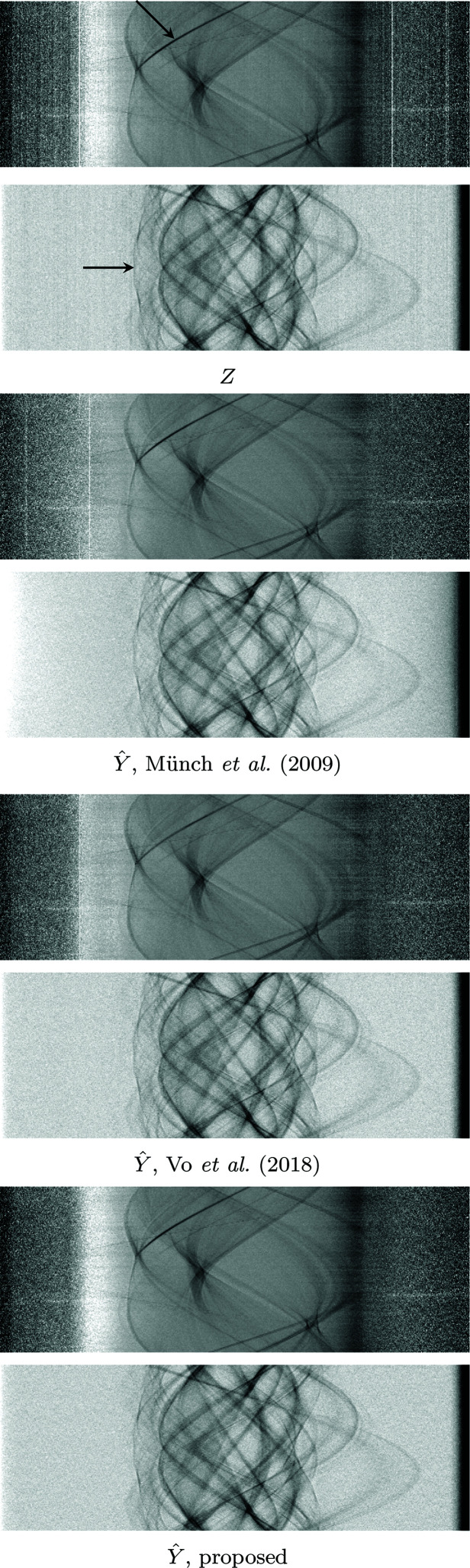
Comparison of two sinograms of *Fly* after different denoising procedures. Top to bottom: noisy sinogram *Z*; Münch *et al.* (2009 ▸) ▸; Vo *et al.* (2018 ▸) ▸; proposed procedure based on BM3D denoising. Although Vo *et al.* (2018 ▸) ▸ is very effective at removing streaks, it also considerably affects the sinogram features; note, for example, the considerably weaker bold diagonal line (indicated by the first arrow) compared with the other algorithms. Both Münch *et al.* (2009 ▸) ▸ and Vo *et al.* (2018 ▸) ▸ also distort larger areas of the sinograms, as pointed out by the second arrow; these problems are absent from the BM3D-based result. Although not visually obvious here, the differences cause severe artifacts in the tomograms, as can be seen in Figure 13 ▸.

**Figure 13:**
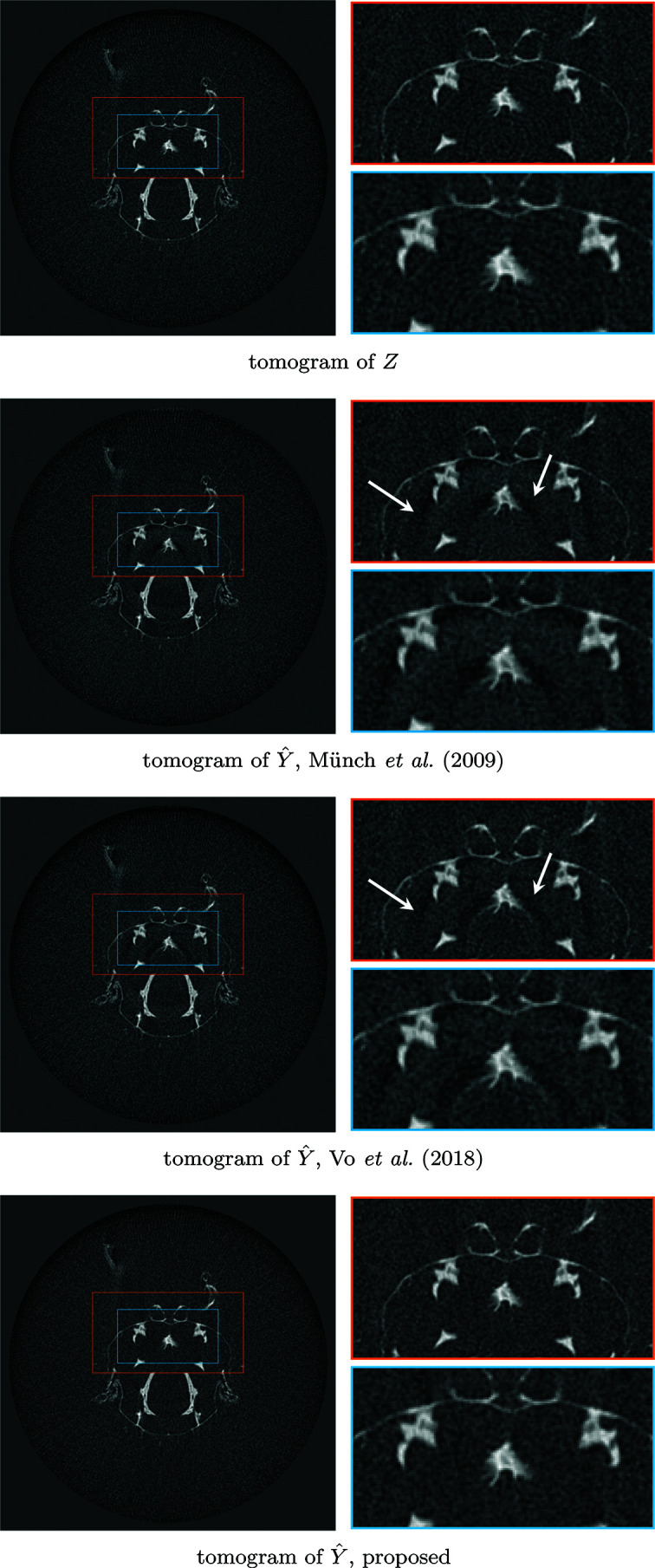
Comparison of resulting tomograms after different denoising procedures on the second sinogram of *Fly* shown in Figure 12 ▸. Top to bottom: noisy reconstructed object; Münch *et al.* (2009 ▸) ▸; Vo *et al.* (2018 ▸) ▸; proposed procedure based on BM3D denoising. Although all methods achieve good results in removing the streaks, both Münch *et al.* (2009 ▸) ▸ and Vo *et al.* (2018 ▸) ▸ introduce strong shadows absent from the proposed estimate, as indicated by the arrows.

The *Fly* dataset consists of 180 projections with 50 s exposure (detector pixel size 27 µm, demagnified to 15.7 µm by cone-beam geometry) collected using a Sigray Prisma X-ray micro-tomography instrument at 34 kV; each sinogram is 512 pixels wide. The *Fly* contains both extreme streaks caused by defective detectors and approximately normally distributed streaks, although they are generally more intense towards the edges of the projections due to weak photon flux and Poisson noise affecting the bright-field. Thus, the *Fly* benefits greatly from both the extreme streak removal procedure of Section 3.4 and relaxing the stationarity assumption by performing PSD estimation and denoising in multiple parts for each sinogram as described in Section 3.3.3. The denoising results of two different sinograms are shown in Figure 12 ▸; the corresponding tomograms of the second sinogram of Figure 12 ▸ are shown in Figure 13 ▸.

**Figure 14:**
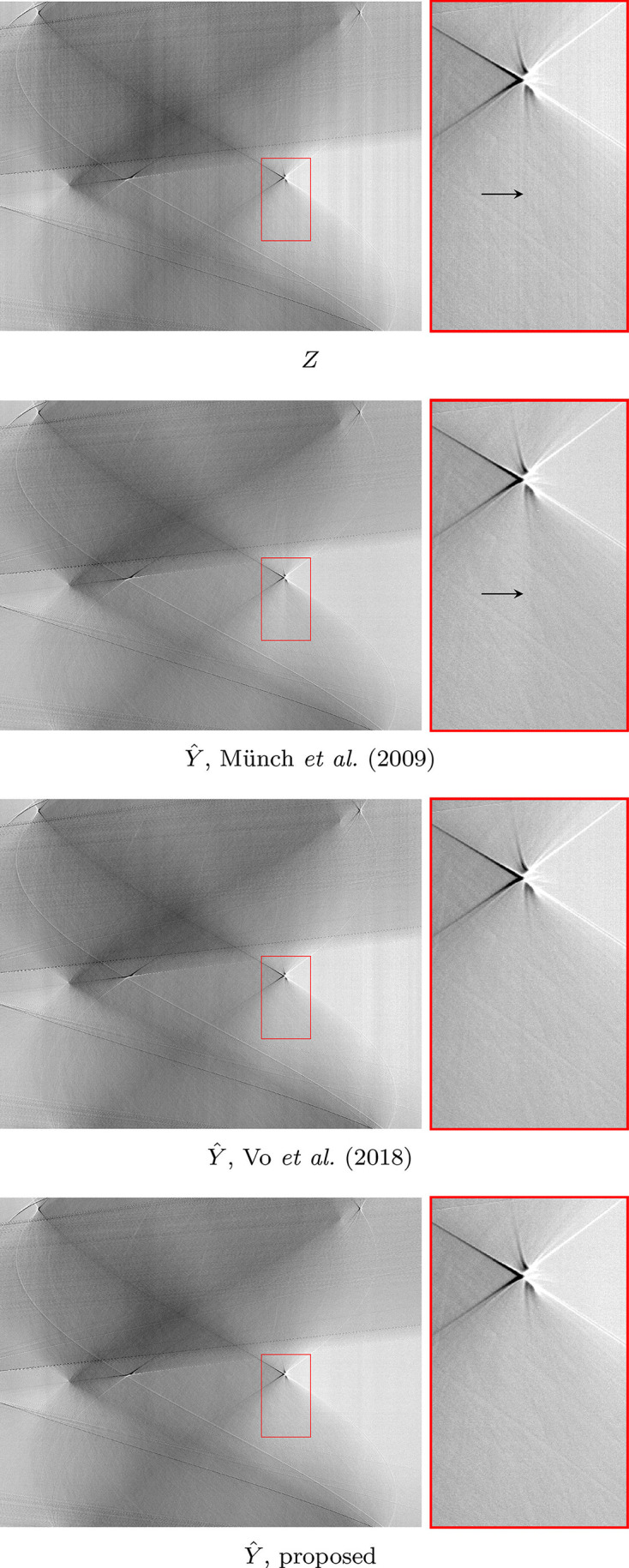
Comparison of sinograms after different denoising procedures on 00076. Top to bottom: noisy sinogram *Z*; Münch *et al.* (2009 ▸) ▸; Vo *et al.* (2018 ▸) ▸; proposed procedure based on BM3D denoising. Note how in the zoom-in the proposed method manages to remove streak noise without creating additional artifacts. Münch *et al.* (2009 ▸) ▸ creates a horizontal streak-like artifacts as seen in the middle of the zoom-in, not present in the noisy sinogram; Vo *et al.* (2018 ▸) ▸ does not fully denoise the sinogram.

**Figure 15:**
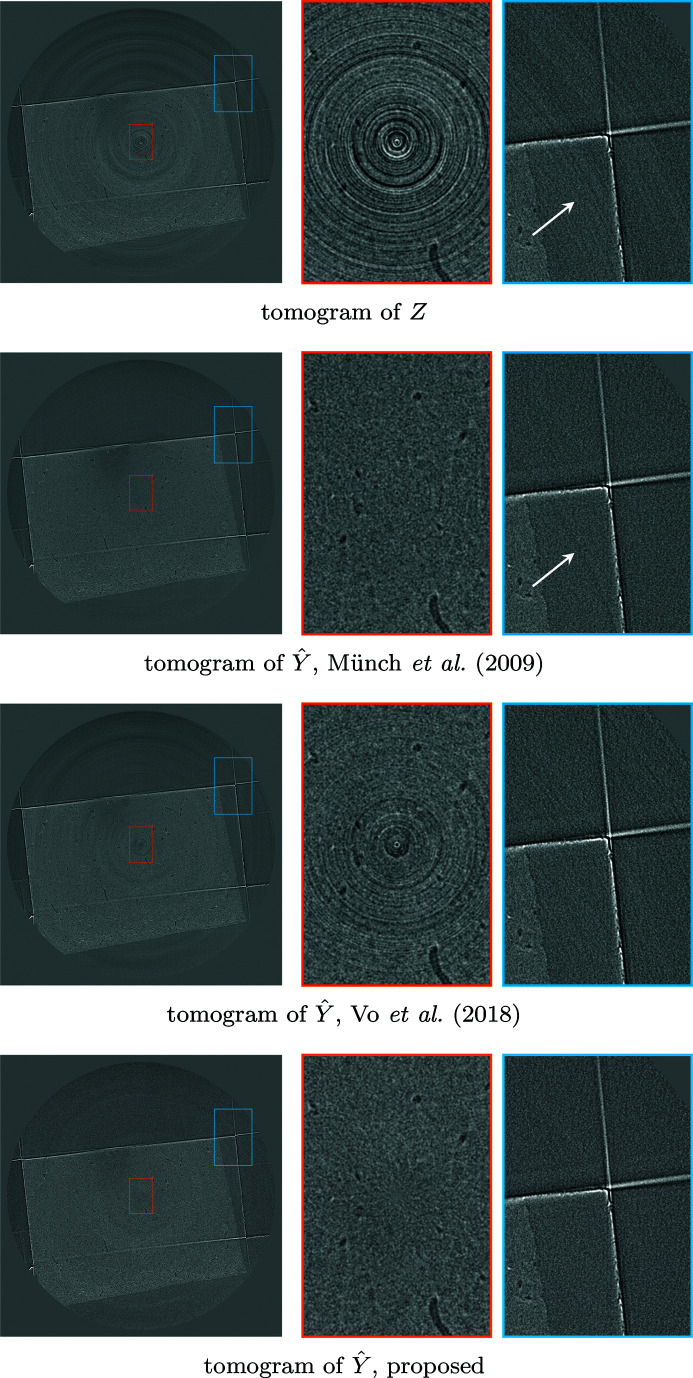
Comparison of resulting tomograms after different denoising procedures on 00076. Top to bottom: noisy reconstructed object; Münch *et al.* (2009 ▸) ▸; Vo *et al.* (2018 ▸) ▸; proposed procedure based on BM3D denoising. Münch *et al.* (2009 ▸) ▸ manage to remove almost all noise in both low and high frequencies, but create artifacts where the original did not have any, as seen from the rightmost zoom. The proposed denoising procedure removes most of the noise [including the wide streaks still present in Vo *et al.* (2018 ▸) ▸], and does not introduce further artifacts. Note also the central pixel, magnified in the middle, which is very dark for both Münch *et al.* (2009 ▸) ▸ and Vo *et al.* (2018 ▸) ▸, whereas the proposed procedure does not leave any visible artifact.

We also test the algorithm on a soft tissue sample 00076 displaying severe ring artifacts freely available in TomoBank (De Carlo *et al.*, 2018 ▸). The data contain 2000 ▸ projections with 2.2 µm pixels, 100 ms exposure time obtained at the Advanced Photon Source, 2-BM beamline; other experimental parameters are an X-ray energy of 60–70 keV, 10 µm LuAG scintillator, and sample-to-detector distance as 90 mm. The sinograms are 2560 pixels in width. Included are ten samples for bright- and dark-fields, which are averaged to obtain a single bright-field and dark-field. The denoising results of a single sinogram are shown in Figure 14 ▸, and the corresponding tomograms in Figure 15 ▸.

### 4.1. Implementation details   

We use the BM3D implementation for Python (available from the PyPI package *bm3d*) with the ‘refilter’ profile and input PSD estimated as described in Section 3.3.2.

For the multiscale denoising procedure, we performed vertical binning with 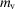 = 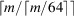 ≃ 64 pixels; this value, being slightly larger than the height of the BM3D search neighborhood of Section 2.2.1 (39 × 39 pixels), allows our method to attenuate streaks which change slowly across the angle – a larger value of *m*_v_ might be used to deal with streaks featuring faster variation. The number of horizontal scales *K* for each sinogram was set as 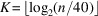, which gives 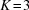 for *Fly* and the Shepp–Logan phantom, and 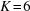 for 00076; these values offer a compromise between denoising wide streaks versus preserving low-frequency signal components – larger values of *K* not only result in *Z*_*K*_ narrower than the BM3D search neighborhood but also in extremely coarse scales that naturally feature a very high SNR that may lead to overestimating ς_*K*_ and hence to oversmoothing. For the localized processing of each scale (Section 3.3.3), we estimate ς_*k*_, 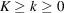 and apply BM3D denoising on 39-pixel wide segments, following the width of BM3D search neighborhood. For the attenuation of extreme streaks, we used a 19 × 19 pixels window.

To consider the computational cost, we note that the full denoising process of *Fly* (181 × 512 × 512 pixels) run single-threaded on an AMD Ryzen 7 1700 processor takes about one hour, mostly due to the BM3D denoising in CPU. A highly parallel GPU-based implementation is expected to reduce this run time to the scale of seconds (Davy & Ehret, 2020 ▸). The complexity of BM3D is linear with the number of pixels in the noisy image. Thus, the computation time is directly proportional to the sinogram height after vertical binning. As each iteration of the horizontal multiscale denoising halves the number of pixels, the computational cost of BM3D for an extra iteration *k* > 0 is then a 1/2^*k*^th of the cost of 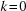, the total for all 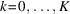 being at most twice that of single scale denoising of *Z*_0_.

The correlation kernels *g*_c_ and 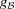 do not depend on the input or scale, and can thus be pre-computed. To compute 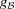, we use directly the definition (23) through a Monte Carlo simulation of sample standard deviation in the Fourier domain. We note that, as the kernel is vertically constant, it suffices to perform this simulation in 1-D and repeat the kernel *m*_v_ times in the vertical dimension.

For Münch *et al.* (2009 ▸) ▸ and Vo *et al.* (2018 ▸) ▸, we use implementations *remove_stripe_fw* and *remove_all_stripe* provided by the *tomopy* Python library of Gürsoy *et al.* (2014 ▸) ▸. The tomograms of each experiment are reconstructed using the *xpack* library (Marchesini *et al.*, 2020 ▸).

## 5. Discussion and conclusions   

We have presented a model for streak noise in the sinogram domain as locally stationary correlated noise additive in the logarithmic scale. Based on this model, we have described a BM3D-based multiscale denoising procedure removing streak noise, and, consequently, the tomogram ring artifacts. The use of the recently proposed variant (Mäkinen *et al.*, 2020 ▸) of BM3D is crucial for this work, as we deal with long-range noise correlation which earlier BM3D designs could not handle satisfactorily.

Tested on both synthetic and real data, our denoising procedure achieves state-of-the-art performance in streak removal. Compared with the two popular streak removal algorithms (Münch *et al.*, 2009 ▸ ▸; Vo *et al.*, 2018 ▸) ▸, our procedure achieves superior results both visually and quantitatively in terms of signal-to-noise ratio. Although all tested algorithms manage to successfully remove most streak noise, both Münch *et al.* (2009 ▸) ▸ and Vo *et al.* (2018 ▸) ▸ tend to create large distortions especially where the intensities of the underlying sinogram columns vary significantly. This type of artifact hinders interpretation of the results and subsequent analysis such as segmentation. In comparison, the proposed algorithm offers similar or better streak removal without further distorting the sinogram.

The proposed multiscale framework, here using basic pixel binning and debinning, could be extended to more sophisticated scale decompositions, such as steerable pyramids, contourlets, or dual-tree wavelets (Kovacevic & Chebira, 2008 ▸). In this work, we have considered each sinogram as a separate input for BM3D for simplicity, but the same mechanism can be used for simultaneous filtering of the entire 3-D stack of sinograms. In this way, the similarities between consecutive sinograms of the stack could be utilized within the collaborative filter.

As the sinograms are commonly processed after a logarithmic transform, we have not discussed inversion of the logarithm needed for denoising. Although the exponential function is naturally the inverse of the logarithm, the non­linearity of the logarithm causes bias in a denoised sinogram if inverted this way. Hence, if the sinogram should be reverted back from the logarithmic domain after denoising, an exact unbiased inverse (Mäkitalo & Foi, 2010 ▸; Mäkitalo *et al.*, 2010 ▸) should be used instead.

## Figures and Tables

**Figure 11:**
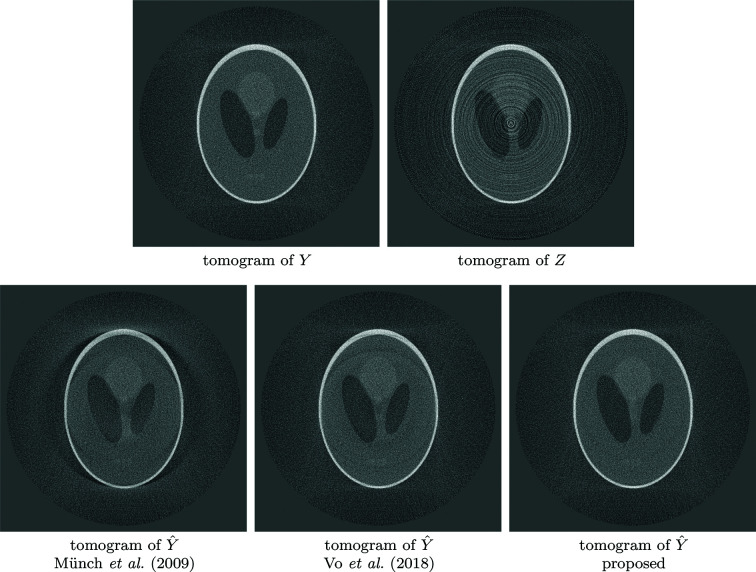
Corresponding tomograms of Figure 10 ▸. Top: 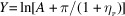 and *Z*. Bottom, left-to-right: Münch *et al.* (2009 ▸) ▸, Vo *et al.* (2018 ▸) ▸, and proposed procedure based on BM3D denoising. Note the strong circular components on both Münch *et al.* (2009 ▸) ▸ and Vo *et al.* (2018 ▸) ▸ which are method artifacts present only in the results by these two algorithms.

**Table 1:** Average signal-to-noise ratio (SNR) after attenuation of streaks in the Shepp–Logan phantom subject to mixed streak and Poissonian noise as in (14), with different combinations of {{\rm{std}}(\eta_{{}_{{\rm{P}}}})} and peak values of *A*, with {\rm{peak}} = \infty being the limiting case for which {\pi\! = \!0} As all of the algorithms aim to remove streak noise only, the SNR values are calculated with Y = \ln[A+\pi/(1\!+\!\eta_{{}_{{\rm{P}}}})] as {{\rm SNR}(\hat{Y}) = 10\,{\rm{log}}_{10}({\rm{svar}}_{X}\{Y^{2}\}/{\rm{smean}}_{X}((\hat{Y}\!-\!Y)^{2})), where svar and smean denote sample variance and sample mean, respectively. Each value of the table is the average SNR over ten different noise realizations.

		SNR			
Peak	std(η_P_)	Noisy	Münch *et al.* (2009 ▸) ▸	Vo *et al.* (2018 ▸) ▸	Proposed
∞ (π = 0)	0.005	32.61	11.80	28.97	44.05
	0.01	26.59	11.78	28.52	39.19
	0.02	20.58	11.72	27.48	34.29
	0.05	12.77	11.49	24.62	27.24
2560	0.005	32.66	11.85	28.32	38.41
	0.01	26.64	11.82	27.89	35.90
	0.02	20.63	11.77	26.90	32.63
	0.05	12.82	11.54	24.21	26.67
1280	0.005	32.71	11.90	27.76	36.51
	0.01	26.69	11.87	27.36	34.31
	0.02	20.68	11.82	26.45	31.55
	0.05	12.86	11.59	23.92	26.21

## Footnotes

1 As *P*_raw_ is a 3-D array, the pixels of *I*_B_ and *I*_D_ are replicated through the angle dimension for the operations in (12).

2 Extreme streak noise arising from defective detectors is addressed separately in Section 3.4.

3 Adopting the kernels (25) with arbitrary values of ς_*k*_ corresponds to assuming η_*k*_ approximately white with variance , which may differ from 2^*k*^std(η_0_) against (21). This assumption becomes increasingly appropriate as *k* grows for non-white η_0_ featuring mild local horizontal correlations, as the binning in  is tantamount to a convolution and decimation, leading to a flattening of the PSD.
